# Reproductive Choices in Haemoglobinopathies: The Role of Preimplantation Genetic Testing

**DOI:** 10.3390/genes16040360

**Published:** 2025-03-21

**Authors:** Georgia Kakourou, Christina Vrettou, Thalia Mamas, Joanne Traeger-Synodinos

**Affiliations:** Laboratory of Medical Genetics, St. Sophia’s, Medical School, Children’s Hospital, National and Kapodistrian University of Athens, 11527 Athens, Greece; cvrettou@med.uoa.gr (C.V.); tmamas@med.uoa.gr (T.M.); jtraeger@med.uoa.gr (J.T.-S.)

**Keywords:** haemoglobinopathies, preimplantation genetic testing, thalassemia, thalassaemia, sickle cell disease, HLA-typing, reproductive choices, PGT-M

## Abstract

Haemoglobinopathies are among the most prevalent genetic disorders globally. In the context of these conditions, preimplantation genetic testing (PGT) plays a pivotal role in preventing genetic diseases in the offspring of carrier parents, reducing the need for pregnancy termination and enabling the selection of compatible sibling donors for potential stem cell transplantation in cases of thalassemia or sickle cell disease. This review explores the evolving role of PGT as a reproductive option for haemoglobinopathy carriers, tracing the development of PGT protocols from patient-specific to comprehensive testing enabled by advanced technologies like next-generation sequencing (NGS). We discuss key technical, biological, and practical limitations of PGT, as well as the ethical considerations specific to haemoglobinopathies, such as the complexity of interpreting genotypes. Emerging technologies, such as whole-genome sequencing, non-invasive PGT, and gene editing, hold significant promise for expanding applications but also raise new challenges that must be addressed. It will be interesting to explore how advancements in technology, along with the changing management of haemoglobinopathies, will impact reproductive choices. It is anticipated that continued research will improve genetic counseling for PGT for haemoglobinopathies, while a careful evaluation of ethical and societal implications is also required. Responsible and equitable implementation of PGT is essential for ensuring that all families at risk can make informed reproductive choices.

## 1. Introduction

### 1.1. Understanding Haemoglobinopathies

Haemoglobinopathies comprise the most prevalent genetic diseases worldwide [1]. It is estimated that 5–7% of the global population is heterozygous for a genetic variant that results in impaired hemoglobin production, leading each year to over 300,000–500,000 affected newborns. Most cases (approximately 350,000) involve sickle cell disease (SCD), while most of the remaining cases are affected by transfusion-dependent β-thalassemia (TDT) (traditionally known as thalassemia major) (https://thalassaemia.org.cy/el/haemoglobin-disorders/, accessed on 7 March 2025). The tropical and subtropical regions of the world, where falciparum malaria is or was endemic, have the highest incidence of individuals with pathological globin alleles due to a selective advantage of heterozygotes [2]. Currently, with population movements, along with the implementation of effective prevention programs in some countries, the epidemiological map for haemoglobinopathies is evolving, such that they are now considered a global public health issue, even in traditionally non-endemic regions of the world [3,4]. For an overview of haemoglobinopathy prevalence, we refer readers to IthaMaps an online resourcethat, through interactive maps, provides country-specific information on prevalence, incidence, disease burden, and globin gene mutation frequencies [5].

Haemoglobinopathies affect the production, structure, or function of hemoglobin (Hb), the principal protein within red blood cells. The role of hemoglobin is to transport oxygen (O_2_) and carbon dioxide (CO_2_) between the lungs and the tissues. There are several types of normal hemoglobin, all of which are tetrameric molecules comprising four polypeptide globin chains, each bound to a heme molecule [6]. Every normal hemoglobin molecule has two globin chains originating from the genes of the α-globin gene cluster on chromosome 16p13.3 *(HBZ* encoding embryonic ζ chains, and *HBA2* and *HBA1* encoding *α* chains), and the other two from the β-globin gene cluster on chromosome 11p15.5 *(HBE* encoding embryonic ε chains, *HBG2* and *HBG1* the fetal γ chains, *HBD* the adult δ chains, and *HBB* the adult β chains). The genes in each cluster are organized in the order of their ontological expression, and the different types of hemoglobin are produced following the transition of gene expression during development, through precise and coordinated control of transcriptional, post-transcriptional, and post-translational mechanisms. Of note is that, although a normal adult has two functional *α*-globin genes on each chromosome 16 (total 4) and a single functional β-globin gene (total 2), the fine regulation of globin gene expression ensures an equal quantity of α-globin chains with β-globin chains, and any imbalance is one of the key pathophysiological mechanisms influencing the degree of clinical severity in patients [7].

Haemoglobinopathies comprise a wide range of disorders. Reduced or absent synthesis of one or more globin chains is characteristic of thalassemia. The thalassemia syndromes are classified according to the globin chain(s) with reduced synthesis. Thus, besides the more common α- and β-thalassemias, there are cases, for example, with δβ-, δ-, γδβ- and very rarely εγδβ-thalassemia. Structural changes in the affected globin polypeptide (and thus Hb molecule) underlie diseases such as sickle cell disease (SCD) or some forms of hemolytic anemia and erythrocytosis. SCD is caused by the most frequent clinically relevant Hb variant, HbS, in homozygosity or in combination with β-thalassemic or certain other β-globin variants. Other β hemoglobin variants common in some populations are often associated with clinically significant manifestations, including Hb E, Hb C, Hb D-Punjab, and Hb O-Arab. Additionally, there are numerous less common and rare Hb variants affecting either the α- and β-globins, most of which do not have any clinical consequences, although a handful have been associated with erythrocytosis or hemolytic anemia of varying severity. Populations in which haemoglobinopathies are endemic usually have more than a single category of variants, and thus the spectrum of clinically expressing patients may include homozygotes, compound heterozygotes, and, in rare cases, patients with complex genotype interactions.

Over 1500 variants involving globin genes have been documented (HbVar: https://globin.bx.psu.edu/hbvar/menu.html, accessed on 7 March 2025, and ITHANET: https://www.ithanet.eu, accessed on 7 March 2025), among which around 500 have potential clinical significance. These genomic variants include deletions that eliminate functional genes and/or their regulatory elements, or nucleotide changes (otherwise known as single-nucleotide variants, SNVs) within or near globin genes that disrupt correct gene expression and/or protein synthesis. Approximately 80% of all known α-thalassemia alleles are attributed to deletions affecting one or both of the duplicated α-globin genes, *HBA1* and *HBA2*. The deletion of both *HBA1* and *HBA2* genes from the affected chromosome completely abolishes the synthesis of α-globin by the affected allele. The remaining 20% are SNVs (also known as non-deletion defects) in either the *HBA1* or *HBA2* genes, rare deletions, some very large and others involving only distal regulatory elements of the α-globin gene cluster and also duplications (as anti 3.7 and 4.2 triplicated α-globin genes) [8,9,10]. In β-thalassemia, variants causing dysfunctional gene expression and/or abnormal protein synthesis are predominantly SNVs (over 95%), while deletions involving the β-globin gene, with or without the cluster regulatory regions, are much less frequent [11,12,13]. In thalassemia, the consequence of variants abolishing the synthesis of globin (null variants) is traditionally presented with a “zero” or “0”, as in α^0^-thalassemia or β^0^-thalassemia and the variant alleles leaving a residual level of globin synthesis are referred to as “plus” variants, as in α^+^-thalassemia or β^+^-thalassemia and β^++^-thalassemia (very low reduction of synthesis, silent β-thalassemia). All structural changes in hemoglobin molecules are caused by missense SNVs [14].

### 1.2. Inheritance and Phenotypic Heterogeneity

Haemoglobinopathies are usually inherited in an autosomal recessive manner, and as mentioned above, numerous globin gene variants have been described. In recent years, the use of new molecular diagnostic approaches, including third-generation sequencing, has facilitated improved diagnostic accuracy as well as the identification of rare globin gene variants [15,16]. The recommended strategy for detecting couples who are at risk for transmitting haemoglobinopathy to their offspring involves an assessment of hematologic findings alongside those of the comprehensive genotyping result [17,18]. However, complexity of genotypes and hematological phenotypes is not uncommon and may present complications when counseling and informing couples. This problem is most acute in cases where the partner of a known carrier remains without an identified genetic variant to justify abnormal hematological findings or is identified with a previously unknown globin gene variant.

Clinically relevant haemoglobinopathies are usually caused by homozygosity or compound heterozygosity for variants in the same globin gene. In β-globinopathies, it can usually be predicted that in carrier couples with β^0^, severe β^+^, δβ variants, δβ-Lepore, and HbS, as well as co-inheritance of HbC, HbΕ, and Hb O-Arab with β^0^ and severe β^+^ variants, there is a 25% chance that their child will be affected by transfusion-dependent thalassemia (TDT) or sickle cell disease (SCD). With other combinations of heterozygosity, such as those with a mild β^++^ variant potentially co-inherited with a β^0^ or severe β^+^ variant, the affected child is likely to have non-transfusion-dependent thalassemia (NTDT, traditionally known as thalassemia intermedia). However, NTDT has a variable clinical phenotype, which may range from mild to moderate anemia with unpredictable transfusion requirements (from none to occasional) and sometimes also other complications. In summary, the correlation of genotype with phenotype in β-globinopathies is often complex and unpredictable.

In α-globinopathies, there is generally a more robust correlation of genotype with underlying phenotype [19]. There are two clinically relevant forms of α-thalassemia. The first is Hb Bart’s hydrops fetalis, usually fatal with perinatal or in utero death (unless subjected to intrauterine blood transfusion), and it is caused by a complete absence of α-globin gene synthesis due to homozygosity of α^0^-thalassemia alleles. Pregnancies affected by Hb Bart’s hydrops fetalis are associated with a risk of maternal health complications as a consequence of carrying a hydropic fetus (toxaemic complications). The other α-globinopathy of clinical significance is known as HbH disease, so-called due to the observation of an abnormal Hb tetramer consisting of only β-globin chains in peripheral blood. HbH disease occurs as a result of reduced α-globin synthesis to levels approximately 25% of normal. Cases with HbH disease have inherited just a single functional α-globin gene (or equivalent), and they usually have clinical presentation of a non-transfusion-dependent chronic moderate life-long anemia. To complicate the picture even further, certain SNVs, usually affecting the *HBA2* gene, may be associated with a more severe HbH phenotype, either in the compound heterozygous state with α^0^-thalassemia alleles or with another SNV in the homozygous or heterozygous state [19].

Finally, it should be noted that variants of the globin genes (*HBA1/2* and *HBB*) and their clusters co-exist in most endemic populations and thus can be co-inherited as complex genotypes. The co-inheritance of genetic variants that reduce α-globin synthesis (such as α-thalassemia deletions) may ameliorate the hematological and clinical phenotype of individuals with homozygous or compound heterozygous β-thalassemia. Rare cases of individuals who are homozygous or compound heterozygous for β-thalassemia alleles (β-thalassaemia major) and possess only a single functional α-globin gene (HbH disease) exhibit a phenotype consistent with non-transfusion-dependent thalassemia (NTDT). In contrast, individuals who are heterozygous for β-thalassemia and inherit additional functional α-globin genes (beyond the normal four) have an aggravated hematological phenotype. Furthermore, individuals with more than five functional α-genes, especially when co-inherited with heterozygous β^0^-thalassemia, may develop thalassemia intermedia of increasing severity, with variable transfusion requirements, depending on the number of extra functional α-globin genes [20]. Although such combinations are relatively uncommon, they can give rise to diverse phenotypic interactions, some of which may be entirely novel and phenotypically unpredictable. Generally, the broad and complex molecular basis of haemoglobinopathies underlies their extreme phenotypic heterogeneity. In some cases, the phenotype may also be influenced by rare variants in cis-acting regulatory elements, as well as trans-acting regulatory processes, including, for example, epigenetic factors and even histone modifications [7,21]. Such parameters are expected to contribute to the variable severity of phenotypes observed in individuals with the same pathological globin genotype, even among members of the same family, although they are rarely investigated or confirmed. Despite international collaborations (e.g., The International Haemoglobinopathy Research Network, INHERENT, (https://inherentnetwork.org/index.php/en/, accessed on 7 March 2025) or the Haemoglobinopathies in European Liaison of Medicine and Science, HELIOS (https://www.cost.eu/actions/CA22119/, accessed on 7 March 2025), as well as useful online tools (e.g., Ithanphen on the Ithanet Portal, https://www.ithanet.eu/db/ithaphen, accessed on 7 March 2025), many challenges remain when trying to predict the potential reproductive outcomes in couples identified with unusual, rare, or novel globin gene variants in the framework of carrier screening programs.

### 1.3. Reproductive Choices

Many countries (like Greece, Cyprus or Iran) have established national programs to prevent haemoglobinopathies, including carrier screening, genetic counseling, and, for high-risk couples, the option of prenatal diagnosis, which have contributed to a decrease in affected births over time [22,23,24,25]. However, access to these services varies considerably between countries or regions due to differences in healthcare infrastructure, resources, and societal conditions. In some regions, religious institutions have historically played a central role in promoting genetic screening and counseling. The most notable example is that of Cyprus, where in 1983, the Church of Cyprus introduced a mandatory premarital screening program requiring couples to obtain a “premarital certificate” confirming carrier status before marriage. This initiative was instrumental in reducing the incidence of thalassemia major by ensuring that carrier couples received appropriate genetic counseling [24,26,27].

The reproductive choices available to carrier couples typically include natural pregnancy, followed by prenatal diagnosis, preimplantation genetic testing (PGT), or use of donor gametes. While options such as not having children or adoption may also be considered, we focus on options directly related to supporting reproductive procedures and pregnancy.

Prenatal diagnosis for haemoglobinopathies is performed on fetal DNA from material obtained by invasive procedures in the first trimester (chorionic villus sampling) or the second trimester of pregnancy (amniocentesis). The discovery of cell-free fetal DNA (cffDNA) in maternal plasma has led to the development of technologies for the non-invasive detection of genetic diseases early in pregnancy (usually from the 10th week of gestation). With such “noninvasive” prenatal procedure, the risks associated with traditional invasive prenatal diagnosis sampling methods are minimized, as analysis is performed on maternal blood following simple blood sampling. Several strategies have been described for non-invasive prenatal diagnosis of haemoglobinopathies; however, it is not yet offered in routine clinical practice [28,29].

Preimplantation genetic testing is an important alternative reproductive option and represents a major advancement in family planning, as it allows for the identification and selection of unaffected embryos prior to implantation. This approach not only reduces the risk of passing on inherited disorders but also avoids the risk of pregnancy loss associated with invasive prenatal procedures or potential pregnancy termination in the case of an affected fetus. The substantial increase in PGT utilization following the widespread implementation of carrier screening programs reflects the preference of couples to make informed reproductive decisions and proactively plan for a healthy family [30].

There is broad consensus on the recommendations for prenatal diagnosis (and consequently PGT) for haemoglobinopathies caused by clinically significant genotype combinations in the *HBA1/2* or *HBB* genes, as outlined in the best practice guidelines of the European Molecular Quality Network (EMQN) [17]. Prenatal diagnosis is generally not recommended for milder forms of haemoglobinopathies, such as those where there is a risk of having a child with thalassemia intermedia or HbH disease, unless specific SNVs in the α-globin genes are involved. In these cases, as well as when the genotype alone cannot predict whether the offspring will have a life-threatening or mild form of globinopathy, PGT may be a more appropriate option.

In this review, we present the current role of PGT as a reproductive choice for haemoglobinopathies. This will involve exploring its effectiveness, evolving protocols in preventing disease transmission within families, as well as technical, ethical, and social limitations, while considering future advancements. Ultimately, the goal is to assess whether PGT offers a viable solution for helping families navigate these complex reproductive decisions and to identify areas where further advancements in protocols and technology could enhance its utility.

## 2. The Evolution of PGT in Addressing Haemoglobinopathies

Preimplantation genetic testing is a process used in assisted reproduction to test embryos for specific genetic conditions before transfer into the uterus. The primary applications of PGT include testing for monogenic diseases (PGT-M), chromosomal abnormalities (structural rearrangements or aneuploidy, PGT-SR or PGT-A, respectively), and mitochondrial DNA variants, all aimed at increasing the likelihood of a healthy pregnancy and birth. More recently, PGT has been applied to polygenic diseases, also sharing the goal of a healthy pregnancy and birth, a practice that has raised ethical and scientific concerns about its efficacy [31,32]. In the case of haemoglobinopathies, PGT has a valuable role in preventing genetic disease in offspring of carrier parents, avoiding pregnancy termination, and enabling treatment of an existing affected child with thalassemia or SCD by selecting a compatible sibling donor for potential future stem cell transplantation (PGT for HLA-typing) [33,34].

PGT-M has evolved through the years, continuously enhancing sensitivity and specificity for diagnosing monogenic disorders. The primary challenge in PGT for haemoglobinopathies arises from the diseases’ genetic complexity and the diversity of affected genotypes. Conventional approaches have involved the development of individualized protocols, each tailored to detect a specific combination of pathogenic variants. The first attempt in analysis of the *HBB* gene, for the purpose of PGT, involved testing of the first polar body (PB) for the genetic defect of sickle cell anemia to support selection of oocytes prior to fertilization. This involved amplification of a 725 bp sequence of the human HBB gene, followed by nested PCR and restriction enzyme digestion [35]. Over the years, numerous protocols have been applied, summarized in Table 1, and notable advancements in practice have occurred to improve genetic diagnosis, while minimizing the risk of damage to the developing embryo [36]. These include selecting the least damaging biopsy stage (moving to cleavage stage, day 3, and then to blastocyst, day 5, biopsy), determining the number of cells to biopsy (one or two cells in cleavage stage embryos or up to ten cells in blastocyst biopsies), using sequential biopsies for cases of failed or incomplete day 3 diagnoses, and improving cell lysis methods. The techniques that followed from the first approach on PBs primarily relied on direct detection of the pathogenic variant via PCR and were later supplemented by the parallel use of polymorphic markers, initially short tandem repeats (STRs) and then also single-nucleotide polymorphisms (SNPs), for indirect genetic testing. These markers helped to enhance diagnostic reliability by addressing issues like allele dropout (ADO) and contamination, related to the small amount of embryonic DNA analyzed. Optimizing protocols involving direct variant detection and marker analysis was laborious and challenging and depended not only on the intricacies of the targeted region but also on the informativity of family and reference samples.

The introduction of whole-genome amplification (WGA) represented a significant advancement, making it possible to analyze minimal amounts of DNA derived from just a few cells and perform multiple reactions from the same biopsy sample and the same WGA product. Although WGA introduced new challenges, such as amplification bias, WGA techniques have been refined over time with new enzymes and strategies to improve the detection of both monogenic disorders and copy number variations [37,38]. WGA technologies such as multiple displacement amplification (MDA), and multiple annealing and looping-based amplification cycles (MALBAC) have more commonly been used for PGT for haemoglobinopathies (Table 1). Another WGA approach, primary template-directed amplification (PTA), has recently shown great promise in enhancing preimplantation genetic testing for monogenic disorders (PGT-M) and remains a promising area for further exploration [39,40].

WGA also enabled the application of high-yield methodologies, such as SNP arrays and next-generation sequencing (NGS) [41,42]. These methods were rigorously validated against established reference protocols, achieving 100% concordance in diagnosing monogenic diseases, including haemoglobinopathies, before replacing the previous standard approaches (as in [43,44,45]). The advent of NGS has facilitated more in-depth genetic analysis of haemoglobinopathies, enabling the detection of a wider spectrum of variants in embryos [46]. Building on the haemoglobinopathy-specific applications detailed in Table 1, NGS, combined with advanced platforms and diverse analytical strategies, now offers broader applicability to a wide range of PGT indications. This has resulted in maximized data acquisition from each sample, along with reduced costs and turnaround time compared to previous techniques. The evolution of technologies, including NGS and SNP arrays, and the development of sophisticated bioinformatic tools for interpreting data from these platforms have significantly improved PGT accuracy and reliability [47,48,49,50,51,52,53,54]. Many laboratories still employ conventional PGT-M approaches [55]. However, comprehensive testing strategies, linked to higher diagnostic efficiency and improved clinical outcomes, are increasingly being used [56]. Today, comprehensive analysis enables the detection of meiotic and mitotic abnormalities, uniparental disomy, ploidy issues, balanced, and unbalanced chromosomal structural rearrangements, segmental imbalances, and supports sample identification through fingerprinting, minimizing the risk of errors [57,58,59,60,61]. More recently, the implementation of whole-genome sequencing-based PGT (10x coverage) using haplarithmisis has demonstrated improved accuracy by increasing the number of genome-wide informative SNPs (achieving a 10-fold increase compared to genotyping-by-sequencing approaches that target specific areas of the genome). This advancement reduces the impact of whole-genome amplification artifacts and sequencing errors. Simultaneously, it shows superior accuracy in the detection of segmental aneuploidies and balanced translocations, facilitates direct pathogenic variant detection, and supports all forms of nuclear and mitochondrial PGT in a single assay [60].

**Figure 1:**
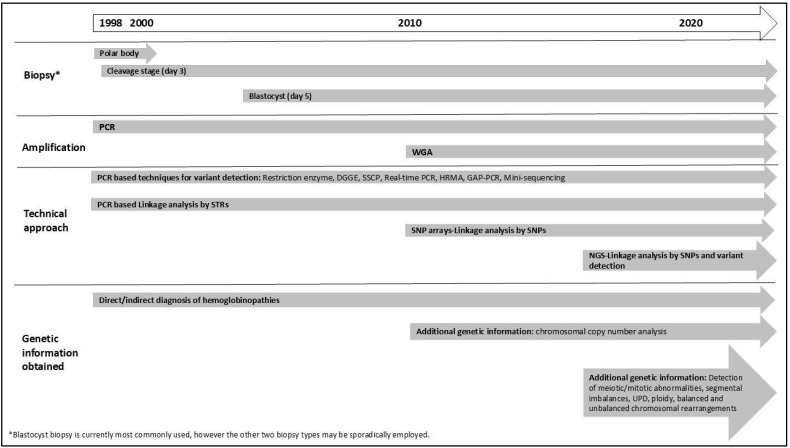
Timeline of the use of PGT for haemoglobinopathies from its initial application to current technologies. DGGE: denaturing gradient gel electrophoresis; HRMA: high-resolution melting analysis, NGS: next-generation sequencing; SNP: single-nucleotide polymorphisms; SSCP: single-stranded conformation polymorphism; STR: short tandem repeat; WGA: Whole Genome Amplification, WGS: whole-genome sequencing. UPD: Uniparental disomy.

In summary, protocols have evolved from patient-specific strategies focusing on detecting a specific pathogenic variant to more generic strategies and a wider scope of analysis. Figure 1 provides a timeline illustrating the major milestones in PGT development. Concurrent testing for all PGT indications (PGT-M, PGT-A, and PGT-SR) has become possible from a single biopsy sample [62]. Studies have demonstrated that combining PGT-M with PGT-A significantly increases pregnancy rates and reduces the risk of spontaneous abortion compared to PGT-M alone. Additionally, research suggests that couples undergoing PGT-M face a similar risk of chromosomal abnormalities as the general population, or can benefit from chromosomal analysis during PGT, highlighting the importance of incorporating PGT-A into PGT-M cycles (Tsuiko O, ESHRE PGT Consortium webinar: “Evolving Technologies in PGT”, November 2023) [63,64]. In the context of haemoglobinopathies, Satirapod et al. (2019) demonstrated the clinical utility of using combined PGT-M and PGT-A to achieve the highest chance of successful implantation and pregnancy in couples at risk of passing on HbE/β-thalassemia; however, the technology used was incapable of detecting mosaicism (see Section 3.1) [65]. Overall, all progressions have made PGT for haemoglobinopathies a reliable, rapid, and clinically effective tool for preventing genetic diseases. Crucially, cross-border collaboration has been essential, particularly for the success of PGT-HLA programs, enabling the entire process from assisted reproduction treatment and embryo selection to pregnancy and ultimately successful hematopoietic stem cell transplantation (HSCT) [66,67].

Undoubtedly, significant progress has been made in PGT protocol development and clinical diagnosis, both in general and specifically in the context of haemoglobinopathies. Laboratories are encouraged to adhere to established recommendations when designing and refining PGT-M protocols, ensuring accuracy and precision in clinical applications [68]. To ensure best practices, these recommendations must be updated based on newer technologies. While maintaining minimum standards, laboratories must also tailor protocols to individual cases, optimizing diagnostic accuracy and incorporating the latest advancements in methodology [69]. Despite these improvements, there remain several additional limitations that need to be addressed (Section 3).

**Table 1:** Evolution of PGT protocols for haemoglobinopathies. (A) Direct analysis of biopsied cells. (B) Protocols based on whole-genome amplification. aCGH: array comparative genomic hybridization; ARMS-PCR: amplification refractory mutation system PCR; DGGE: denaturing gradient gel electrophoresis; FRET: fluorescence resonance energy transfer; HRMA: high-resolution melting analysis; MALBAC: multiple annealing and looping-based amplification cycles; MDA: multiple displacement amplification; NGS: next-generation sequencing; SNP: single-nucleotide polymorphisms; SSCP: single-stranded conformation polymorphism; STR: short tandem repeat; WGS: whole-genome sequencing.

A. Direct Analysis of Biopsied Cells					
Single Indication					
	Biopsy Stage	First Processing	Subsequent Procedures		References
			First Step	Follow-Up Experiments and Analyses	
β-thalassemia/ sickle cell anemia	1st and 2nd polar body	Freeze/heating protocol	Multiplex PCR	STRs (linked/non-linked), nested PCR, and restriction digestion	[70]
	Day 3	Alkaline lysis (in some following freezing)	PCR	Nested PCR, restriction digestion, and reverse dot blot	[71]
				Nested PCR, restriction digestion	[72]
				Two-step ARMS-PCR	[73]
				Real-time PCR with FRET probes	[74]
			Multiplex PCR	Nested PCR, mini-sequencing	[75]
				Nested PCR, mini-sequencing, single STR analysis	[76]
		Proteinase K	PCR	Nested PCR, DGGE	[77,78,79]
				STR analysis	[80]
				Nested PCR, SSCP analysis	[81]
			Multiplex PCR	Nested PCR, SSCP, STR analysis	[82]
			Multiplex PCR	Real-time PCR (analysis of all common b-globin genotypes) and STR analysis to monitor contamination	[83]
			Multiplex PCR	Mini-sequencing, STR analysis	[84]
β-thalassemia/ HBE disease	Day 5	Proteinase K	Multiplex (fluorescent) PCR (one STR)	Mini-sequencing	[85]
α-thalassemia syndromes	Day 3	Proteinase K	Multiplex (fluorescent) PCR	STR analysis (three STRs)	[86]
				GAP-PCR for -SEA detection, internal control fragments, and single STR analysis	[87]
		Alkaline lysis	Multiplex (fluorescent) PCR	STR analysis (three STRs)	[88]
				STR analysis (nine STRs)	[89]
	Day 5 (and day 3)	Proteinase K	PCR	Nested PCR, digital PCR	[90]
HLA-typing	Day 3	Proteinase K	Multiplex PCR	STR analysis	[91]
**Multiple indications**					
β thalassemia/ HLA	Day 3	Alkaline lysis	Multiplex PCR	Mini-sequencing HLA typing and variant analysis, STR haplotying	[92]
				Restriction enzyme digestion and STR HLA haplotyping	[93]
			Multiplex (fluorescent) PCR	Real-time nested PCR, HRMA, STR analysis	[94]
		Proteinase K	Multiplex (fluorescent) PCR (two rounds)	STR analysis	[95] [96]
	Day 3	Alkaline/PK	Multiplex PCR	SinglePlex PCR, mini-sequencing	[66,92] (rebiopsy day 5)
				Restriction enzyme digestion, polyacrylamide gel electrophoresis, STR analysis	[97,98]
β/α-thalassemia/ HLA/aneuploidy	PB/Day 3	Alkaline/PK	Multiplex PCR	Restriction enzyme digestion, polyacrylamide gel electrophoresis, STR analysis	[99,100]
β thalassemia with HLA typing or aneuploidy screening or sex selection (or combination of two-three indications)	Day 3	Proteinase K	Multiplex PCR	Nested PCR, Sanger sequencing, STR analysis	[101,102]
-β-thalassemia, sideroblastic anemia, HLA typing	Day 3 biopsy	Alkaline lysis	Multiplex (fluorescent) PCR	Real-time nested PCR, HRMA, STR analysis	[103]
**B. Whole-genome amplification protocols**					
**Single indication**					
β-thalassemia/ sickle cell anemia	Day 3	MDA	Separate PCRs	Single-cell analysis of two codons of β-globin gene	[104]
	Day 3 and day 5		Targeted re-amplification with multiplex PCR for entire *HBB* locus (coding region and splice junctions) and 17 SNPs	NGS	[46] (also directly on single cells)
	Day 5		Multiplex PCR	STR haplotyping	[105]
			Single-plex PCR and long-segment PCR Or multiplex PCR	Reverse dot blot, SNP or STR analysis	[106] [107]
a-thalassemia syndromes	Day 5	MDA	PCR + NGS	GAP-PCR and SNP haplotyping	[108]
			Multiplex fluorescent PCR	Fragment analysis	[109]
					[110]
		MALBAC and MDA	PCR	GAP-PCR, Sanger sequencing, SNP haplotyping	[111]
**Multiple indications**					
α&β-thalassemia	Day 3	MDA	PCR	GAP-PCR, reverse dot blot and STR analysis	[112]
β-thalassemia/ HLA	Day 3	MDA	Multiplex PCR	STR analysis Sanger sequencing and sequence-specific primer technique for HLA typing	[113,114]
β-thalassemia/ sickle cell anemia/ aneuploidy	Day 3 or day 5	MDA	SNP array	Karyomapping	[43]
	Day 3 or day 5	MALBAC and MDA	Low-coverage WGS and amplification of *HBB* variants and SNPs	NGS	[115]
	Day 5	DOPlify with target sequence enrichment	PCR for pathogenic variant and SNP markers. Target-enriched PCR pooled back into initial WGA product	NGS	[116]
		MDA	Multiplex PCR for SNP enrichment and direct mutation detection	NGS-OneGene PGT	[44]
		MDA	low-pass whole-genome sequencing (WGS) for genomic imbalance and high-depth target enrichment sequencing for HBB variants and SNPs	WGS	[117]
β-thalassemia/ HemoglobinE /aneuploidy	Day 5	MDA	SNP array	Karyomapping	[118]
		PicoPlex or MDA	Multiplex PCR aCGH NGS	Mini-sequencing, STR analysis, chromosomal analysis	[65]
β-thalassemia/ HLA/ Aneuploidy	Day 3/ day 5	MDA	Multiplex PCR	Restriction enzyme digestion, polyacrylamide gel electrophoresis, STR analysis, aCGH	[119]
	Day 3	MDA	All-in-one target region sequencing (WGS)	NGS	[120] (retrospective study)
	Day 5–6	MDA	SNP array	Karyomapping	[45]
			Restriction-site-associated DNA sequencing	HaploPGT algorithm analysis	[47]
HLA/aneuploidy	Day 5	Not specified	aCGH, SNP array	Chromosomal analysis, karyomapping	[121]
α- and/or β-thalassemia/aneuploidy	Day 5 or 6	MDA	PCR for β,α thalassemia and SNP analysis	NGS	[122]
			GAP-PCR/SNP arrays	Detection of SEA-type α-thalassemia by PCR, haplotype analysis and aneuploid screening	[123]
α- and β-thalassemia/HLA typing/aneuploidy	Day 5	MDA	Targeted NGS for pathogenic variant detection (*HBB*, *HBA1*, *HBA2*) and SNP analysis	NGS	[124,125]

## 3. Remaining Challenges in PGT for Haemoglobinopathies: Technical, Ethical, and Accessibility Barriers

### 3.1. Technological/Biological Limitations of PGT

One major challenge in PGT today is chromosomal mosaicism, which has often been inaccurately identified and interpreted over the years, leading to misinformed reproductive decisions and a reduction in the number of embryos available for transfer, sometimes inappropriately. Though mosaicism primarily affects chromosomal assessment rather than the monogenic condition itself, it remains a significant limitation in all PGT practice, especially as the current aim is to offer a universal test for all indications by combining, for example, PGT-M with PGT-A. Mosaicism in PGT has been extensively investigated and discussed, resulting in a substantial body of literature documenting its prevalence and potential impact. Many professional societies have subsequently published recommendations on approaching mosaicism in PGT, offering guidance on detection, interpretation, and counseling [126,127]. Despite these valuable contributions, the inherent complexity of mosaicism continues to pose a challenge in clinical practice, complicating reproductive decision-making.

In addition to the challenges posed by mosaicism, the practical requirement to deduce parental haplotypes by testing family members (or, if possible, by single sperm analysis, if family is unavailable) adds complexity and cost to the process. While some laboratories have attempted to overcome this by testing embryos from the same cycle to support linkage analysis, this approach introduces its own uncertainties due to the unpredictable number and genotypes of available embryos [128]. Moreover, several technical limitations continue to hinder the diagnostic accuracy of PGT across all indications, which is particularly relevant as referrals may involve more than one monogenic condition (in addition to haemoglobinopathies discussed here). These challenges include areas with low-confidence haplotypes, repetitive regions, pseudogenes, and high GC content during sequencing, as well as the difficulty of detecting submicroscopic deletions and tandem duplications. Newer technologies promise to address some of these limitations [129,130,131]. In addition to the above, recombination events and chromosomal aneuploidy can complicate accurate diagnosis, while poor embryo quality or unsuccessful sample amplification can lead to inconclusive or no results. Therefore, PGT outcomes may not always align with expectations.

### 3.2. Ethical Considerations in PGT for Haemoglobinopathies

Despite being among the first group of hereditary diseases to be characterized genetically, haemoglobinopathies, including thalassemia and sickle cell syndromes, remain of high interest for genetic research and present significant challenges for genetic counseling. As discussed in Section 1.2, the complex genetic heterogeneity, varying phenotypic expressions, and the influence of numerous factors on disease severity make it difficult to accurately predict the outcomes of specific gene variant combinations on offspring in some cases. These challenges, along with broader ethical considerations, such as the expanding scope and diagnostic capabilities of PGT and the varying ethical perspectives across different societies, highlight the importance of comprehensive genetic counseling for couples considering PGT for haemoglobinopathies. Couples should have the right to receive counseling to make informed decisions about whether to pursue prenatal or preimplantation genetic testing or to prepare psychologically for the potential challenges of raising a child with a mild form of haemoglobinopathy. This includes but is not limited to conditions such as deletional HbH disease, homozygosity/compound heterozygosity for variants of the β-globin gene, like mild β^+^, β^++^, or even double heterozygosity for more than five α-globin genes and β variants.

In our experience, as a Mediterranean country, there are couples that express a strong interest in avoiding the birth of a child with any haemoglobinopathy. This preference often leads couples to choose PGT as an ethically more acceptable option to minimize the risk of transmitting these disorders, rather than terminating a pregnancy after prenatal diagnosis for a severe form of haemoglobinopathy. Additionally, for couples with a risk of having a child with a mild haemoglobinopathy, for which prenatal diagnosis is not traditionally recommended, PGT may be the only available option to prevent the birth of an affected child. PGT for haemoglobinopathies may also serve a dual role of preventing disease transmission, while also addressing potential fertility challenges associated with hemoglobin disorders, that may arise in a couple where one partner is affected and the other a carrier. In addition to fertility concerns, these couples face a lower likelihood of identifying unaffected embryos, as only 50% rather than 75% of conceptions will be potentially unaffected [132,133].

Despite the benefits, the use of PGT has sparked ethical debates, including concerns about selecting embryos based on milder disease variants, using HLA-matched embryos for therapeutic purposes (which raises concerns about embryo instrumentalization, where embryos are selected primarily for their potential to serve as a source of treatment for another child), and broader issues related to reproductive autonomy. As advancements in treatments for haemoglobinopathies continue, discussions also involve finding the right balance between prevention and treatment. These ethical concerns, along with ongoing disparities in access to healthcare services (see Section 3.3), will continue to influence the future of reproductive choices for families affected by haemoglobinopathies. To ensure patients can make informed reproductive health choices, it is crucial to address the limited awareness and acceptability of reproductive options, provide education, and continuously improve existing comprehensive genetic databases for haemoglobinopathies [134]. This relies on strong collaboration among scientists to expand and refine these databases, thereby supporting clinicians and patients, strengthening genetic counseling, and facilitating the establishment of well-defined standards for a robust ethical framework for PGT in haemoglobinopathies.

### 3.3. Accessibility and Cost Barriers in PGT-M and Overall Cost-Effectiveness

In terms of accessibility and cost, the widespread use of PGT remains restricted, and highly unequal due to financial, infrastructural, and policy-related barriers, particularly in low- and middle-income countries [135]. A recent study by Combs et al. (2023) found that only 24% of at-risk couples in sub-Saharan Africa have access to PGT, primarily due to financial constraints [136,137,138,139]. Even in high-income countries, disparities persist due to variations in insurance coverage and regulatory policies. While some European nations provide financial support for PGT-M through public healthcare systems if couples meet specific criteria, others impose strict regulations that limit accessibility. In the United States, PGT-M is rarely covered by insurance, leaving most couples responsible for the full cost. Beyond direct procedural costs, the high cost of PGT procedures, particularly with advanced technologies such as NGS and haplotype linkage analysis, and the need to test multiple embryos from the same or different IVF/PGT cycles, significantly limit access for many couples. This financial burden, coupled with the lack of adequate infrastructure in certain regions, exacerbates the inequity in access to reproductive technologies, reducing reproductive options for families at risk of haemoglobinopathies.

Despite these challenges, several investigators have supported the cost-effectiveness of assisted reproduction with PGT-M compared with the lifetime treatment costs of affected individuals. A recent systematic review by Nadgauda et al. (2024) analyzed eight cost-effectiveness studies (2008–2023) and found that all concluded favorably for PGT-M in disease prevention [139,140,141,142]. Combs et al. (2023) further compared the cost of IVF/PGT-M for sickle cell carriers to the lifetime treatment cost of an affected individual within the U.S. healthcare system, concluding that PGT-M is the most cost-effective strategy [139]. A recent study presents the most cost-effective strategies for parents seeking an HLA-matched sibling donor, via PGT, to cure an affected child via bone marrow transplantation.

However, beyond financial considerations, the emotional burden on families remains difficult to quantify. A fully informed reproductive decision requires evaluating not only economic factors but also ethical, clinical, and emotional dimensions. To reduce the global burden of haemoglobinopathies, expanding insurance coverage, integrating PGT into public health programs, and developing lower-cost diagnostic strategies are essential. Further research and collaborative efforts are needed to make PGT more accessible and affordable for all couples at risk.

Notwithstanding these challenges, PGT remains a valuable tool for reducing the transmission of haemoglobinopathies. Continued progress in genetic counseling, technological advancements, and the improvement of comprehensive genetic databases, alongside efforts to enhance affordability and ethical frameworks, will be key to making PGT more effective and widely accessible.

## 4. Future Advances in Preimplantation Genetic Testing (PGT)

It is anticipated that more comprehensive and precise tools for preventing the transmission of genetic disorders will be available in the future. However, with these advancements, PGT may also become more complex. The introduction of technologies such as whole-genome sequencing (WGS) for embryos promises to provide a more complete genetic picture. In addition, innovations such as non-invasive PGT may eventually eliminate the need for embryo biopsies. Furthermore, gene editing at the preimplantation stage could provide a potential alternative to traditional PGT by correcting genetic variants directly. These advancements, alongside emerging therapies like gene therapy for haemoglobinopathies, will shape the future landscape of PGT, potentially influencing its role in reproductive choices.

### 4.1. Whole-Genome Sequencing: Expanding the Horizons of PGT

The future of PGT is set to be revolutionized by the introduction of whole-genome sequencing (WGS) for embryos. Unlike traditional genetic testing methods, which typically focus on specific variants, WGS enables the analysis of the entire genome, providing a comprehensive view of both heritable and non-heritable genetic information and allowing for the detection of a range of different abnormalities. This includes the detection of *de novo* variants that occur for the first time in an embryo and are not inherited from either parent. WGS has already been shown to improve PGT accuracy and expand the scope of genetic screening in embryos. However, its widespread adoption remains limited by bioinformatics challenges. Handling large sequencing datasets requires robust computational infrastructure for efficient storage and processing, as well as sophisticated tools for accurate variant detection, haplotype reconstruction, and quality control. Many laboratories rely on commercial software due to limited in-house expertise, restricting flexibility, and standardization. Deep learning models, as highlighted by Alharbi and Rashid (2022), show promise for improving variant interpretation, though their clinical application is still evolving [143]. To fully realize the potential of WGS-PGT, developing validated bioinformatics pipelines with integrated deep learning and standardized reporting will be essential for enhancing accuracy and clinical reliability.

While WGS provides couples with more detailed genetic data, it also complicates decision-making. Couples will be faced with not only inherited genetic risks but also the uncertainty surrounding de novo mutations, which could lead to previously unanticipated health conditions. This increased complexity will require more extensive genetic counseling to help couples understand the implications of these findings and will likely push the boundaries of informed decision-making in reproductive care [144].

### 4.2. Non-Invasive PGT: Reducing Risk and Improving Access

One of the key challenges with traditional PGT is the need for embryo biopsy, an invasive procedure. Several studies have explored the impact of embryo biopsy (polar body, cleavage stage, blastocyst biopsy) on embryo quality [145,146,147,148]. Currently, blastocyst biopsy is generally considered to pose a lower risk to embryo development compared to earlier stages [149,150]. Embryo biopsy is associated with increased costs due to the need for specialized equipment and highly trained personnel. The procedure is also time-consuming, adding to the workload in IVF labs. These challenges make PGT more complex, further underscoring the potential advantages of non-invasive alternatives. Non-invasive PGT could help make genetic testing more accessible, offering a less costly, less risky, and more efficient option for a broader range of couples.

Non-invasive preimplantation genetic testing (niPGT) has been a significant area of research since 2013, when early studies first identified the presence of embryonic DNA in embryo culture media and the blastocoel cavity [151,152]. Since then, numerous studies have primarily focused on optimizing niPGT for chromosomal analysis (PGT-A), exploring various strategies to improve its accuracy and reliability. The findings suggest that refining embryo handling, particularly to minimize maternal DNA contamination, and optimizing the timing of media collection, can enhance niPGT-A performance. However, further research and optimization are still required before it can reliably replace invasive biopsy procedures in routine clinical practice.

While significant advancements have been made in niPGT for PGT-A, the application of non-invasive techniques for monogenic disorders remains limited. Several studies have explored non-invasive genetic testing for thalassemia using cell-free DNA from spent embryo culture medium. Wu et al. (2015) demonstrated that this method, when performed on day 5, can accurately diagnose α-thalassemia-SEA with high efficiency [153]. Liu et al. (2017) confirmed that cell-free DNA in the medium can be amplified to detect thalassemia variants, although diagnostic concordance with biopsied cells remains a challenge [154]. Ou et al. (2022) showed that incorporating blastocoelic fluid (BF) into the culture medium improved the detection rates for β-thalassemia, but issues like DNA fragmentation and contamination persist [155]. While these findings are promising, further research and validation are needed before non-invasive PGT-M can be reliably integrated into clinical practice. While chromosomal analysis is important for optimizing embryo selection, addressing inherited genetic disorders through targeted testing is even more crucial, as it directly prevents the transmission of life-altering conditions. This approach holds great promise and warrants further investigation.

### 4.3. Gene Editing: A Revolutionary Alternative to PGT?

As gene editing technologies like CRISPR-Cas9 continue to evolve, there is growing interest in their potential application at the preimplantation stage. Gene editing offers a revolutionary approach to correcting pathogenic variants directly, potentially eliminating the need for PGT altogether. Instead of identifying embryos that carry harmful genetic variants, gene editing could enable the correction of these variants before implantation, offering a more proactive approach to preventing genetic disorders. Gene editing may help address the genetic challenges that PGT cannot overcome, for example, cases where both partners are affected by a severe haemoglobinopathy and all their offspring are expected to be affected. However, gene editing is still in its early stages and faces significant technical, ethical, and regulatory hurdles. Issues such as off-target effects, the ethical concerns of germline modification, and the long-term consequences of gene editing for future generations will need to be addressed before this technology can be widely adopted [156]. If successful, gene editing could fundamentally alter the role of PGT, providing a new avenue for preventing the transmission of genetic disorders.

### 4.4. Advancements in Haemoglobinopathy Treatment: Impact on PGT

The management of haemoglobinopathies has seen remarkable advancements, including enhanced multidisciplinary care and improved success of hematopoietic stem cell transplantation (HSCT), as well as the development of novel treatments like gene therapy, which are set to significantly impact the role of PGT [14,157]. Gene therapy aims to correct the underlying genetic variants responsible for disorders such as sickle cell disease and β-thalassemia, offering the potential for a cure. These therapies, as they become more effective and accessible, may affect the demand for PGT. While gene therapy could decrease the need for PGT in some cases, it may also complement it in situations where PGT-M identifies only affected embryos, providing an additional option for families and helping avoid embryo wastage.

Gene therapy for haemoglobinopathies has shown significant promise in recent years, but is still in the experimental phase. It focuses on either adding a functional copy of the gene (gene addition) or directly modifying the defective gene (gene editing) to correct the underlying genetic defect. Most therapies involve modifying a patient’s stem cells outside the body (ex vivo) before re-infusing them, using viral vectors such as lentiviruses to deliver the therapeutic genetic material. Currently, clinical trials targeting sickle cell disease and thalassemia have demonstrated promising results [14]. However, the widespread application of gene therapy for haemoglobinopathies is hindered by several challenges, including the very high cost of treatment, the need for specialized centers and skilled personnel, and concerns regarding the long-term efficacy and potential side effects, with data still limited.

Despite these hurdles, the future of gene therapy for haemoglobinopathies is promising. As technology advances, we can expect improvements in cost reduction, treatment efficacy, and broader access to these therapies. It is essential to maintain rigorous oversight and establish strong regulatory frameworks to ensure the safety and efficacy of these methods.

## 5. Conclusions: A Complex but Promising Future for PGT

PGT has emerged as a significant advancement in reproductive medicine. PGT for haemoglobinopathies is crucial, especially in countries where haemoglobinopathies are prevalent, offering couples the opportunity to prevent the transmission of these inherited diseases. This aligns with the goals of preconception care and screening, which aim to identify and reduce reproductive risks before conception. However, while PGT holds immense promise, its implementation necessitates careful consideration of ethical dilemmas and the inherent complexities associated with predicting disease severity based on genetic information alone.

The future of preimplantation genetic testing is filled with promise, offering families more precise and comprehensive tools for making informed reproductive decisions. However, as technology advances, so too does the complexity of these decisions. Whole-genome sequencing, non-invasive techniques, gene editing, and new treatments for haemoglobinopathies will undoubtedly shape the future of PGT, providing more options for couples while also presenting new challenges. Navigating these advancements will require ongoing research, improved genetic counseling, and careful consideration of the ethical, emotional, and societal implications of these technologies. The continued evolution of PGT will ultimately provide families with greater control over their reproductive futures, but it will also require careful, thoughtful application to ensure that these advancements are used responsibly and equitably.
